# Supplementary material for: Chiral multi-curved shell metamaterials integrating compression-torsion and buckling mechanisms for ideal energy absorption
Source: Nat Commun. 2025 Dec 12;16:11359. doi: 10.1038/s41467-025-66443-y (PMC12727706; doi:10.1038/s41467-025-66443-y)
Supplement: Supplementary file 1 — Supplementary Information [file 41467_2025_66443_MOESM1_ESM.pdf]

Supplementary Information

Chiral multi-curved shell metamaterials integrating compression-torsion and buckling mechanisms for ideal energy absorption

Chen-Xu Liu[1], Yizhi Zhang[1], Xinghao Wang[1], Gui-Lan Yu[2], Zhuo Zhuang[1], and Zhanli Liu[1*]

1 Applied Mechanics Lab., Department of Engineering Mechanics, School of Aerospace, Tsinghua University, 100084 Beijing, China
2 School of Civil Engineering, Beijing Jiaotong University, Beijing 100044, China

*Corresponding author, email: liuzhanli@mail.tsinghua.edu.cn

This PDF file includes:

Supplementary Notes 1 to 15

Supplementary Figures 1 to 18

Supplementary Tables 1 to 6

Supplementary Note 1. The main structure of the chiral multi-curved shell (CMCS) metamaterial.

As shown in Supplementary Figure 1, given the shape of the bottom plane and the rotation angle as well as location of the top plane, the main structural configuration can be determined. Each line segment of the bottom polygon, when swept along the rotating ascending path, namely the line from p_i to $\hat{p}_i$ $(i = 1,2,3 \cdots)$, forms the curved surface of the metamaterial. The construction method for the multiple curved surfaces of the outer and inner ring polygons is identical. The area enclosed by these generated curved surfaces forms the main structure of the chiral multi-curved shell (CMCS) metamaterial.

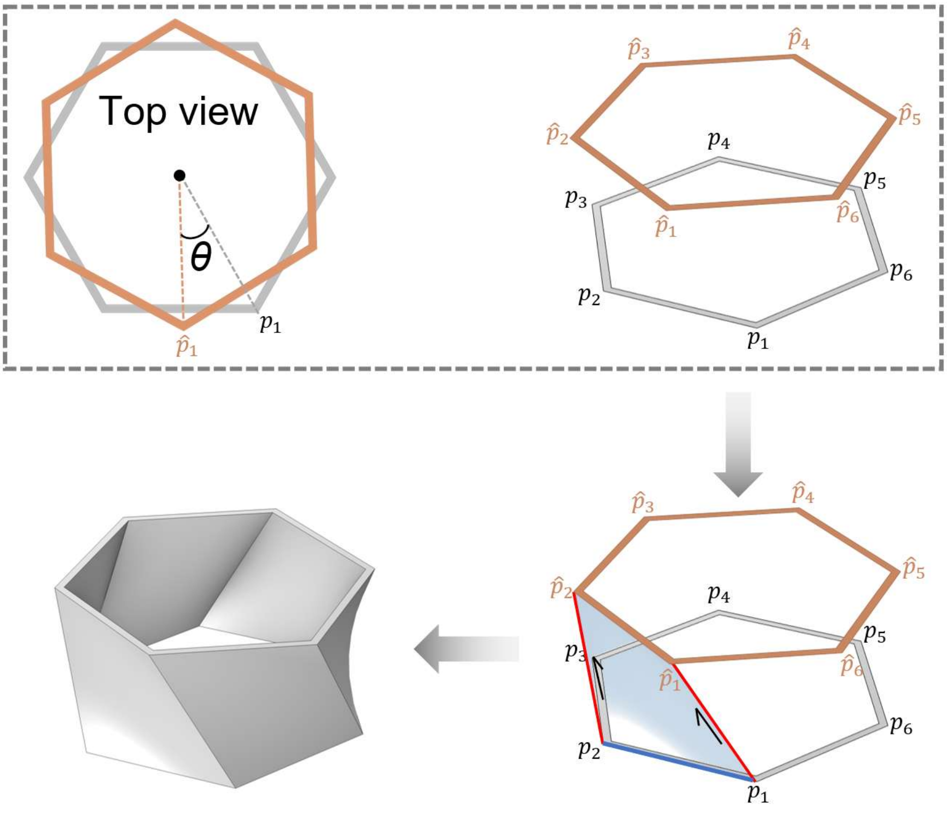

Supplementary Figure 1. Geometry construction method for the main structure of the chiral multi-curved shell (CMCS) metamaterial. θ is the rotation angle of the top plane relative to the bottom plane; p_i $(i = 1,2,3 \cdots)$ is the vertex of the polygon on the bottom plane, and $\hat{p}_i$ $(i = 1,2,3 \cdots)$ is that on the top plane.

Building on the above operations, the main structure of the CMCS metamaterial can be described by analytical equations.

Assume that the center of the bottom polygon is at the coordinate origin, and the two endpoints of an outermost bottom line are represented by $(x_1, y_1, 0)$ and $(x_2, y_2, 0)$, respectively. These coordinates are known quantities, determined by the geometric parameters n, a, and t of the main structure, as illustrated in Figure 1a. The two endpoints of other lines on the bottom surface that are parallel to the outermost bottom line can be expressed as $((1 - 2\tan(\pi/n)\tilde{t}/a)x_1, (1 - 2\tan(\pi/n)\tilde{t}/a)y_1, 0)$ and $((1 - 2\tan(\pi/n)\tilde{t}/a)x_2, (1 - 2\tan(\pi/n)\tilde{t}/a)y_2, 0)$, respectively, $0 < \tilde{t} \leq t$, where $\tilde{t}$ is the distance from the current line to its corresponding outermost line. Hence, the lines on the bottom that parallel to the outermost bottom line can be described by the following equations:

$$\begin{cases} x \in \left[\min\left(\left(1 - \frac{2\tan\left(\frac{\pi}{n}\right)\tilde{t}}{a}\right)x_1, \left(1 - \frac{2\tan\left(\frac{\pi}{n}\right)\tilde{t}}{a}\right)x_2\right), \max\left(\left(1 - \frac{2\tan\left(\frac{\pi}{n}\right)\tilde{t}}{a}\right)x_1, \left(1 - \frac{2\tan\left(\frac{\pi}{n}\right)\tilde{t}}{a}\right)x_2\right)\right] \\ y = \frac{y_1 - y_2}{x_1 - x_2}\left(x - \frac{a - 2\tan\left(\frac{\pi}{n}\right)\tilde{t}}{a}x_1\right) + \frac{a - 2\tan\left(\frac{\pi}{n}\right)\tilde{t}}{a}y_1, 0 \leq \tilde{t} \leq t \end{cases} \tag{S1}$$

As a bottom line sweeps from the bottom to the top, its height can be expressed as:

$$\tilde{b} = \frac{b}{\theta}\tilde{\theta} \tag{S2}$$

where b and θ are known geometric parameters of the main structure, as shown in Figure 1a, corresponding to the ultimate height and rotation angle of a bottom line, respectively; $\tilde{b}$ is the height variable corresponding to the change of the rotation angle variable $\tilde{\theta}$.

The coordinates of the two endpoints of a line on the main structure parallel to the bottom plane can be expressed as $((1 - 2\tan(\pi/n)\tilde{t}/a)(x_1\cos\tilde{\theta} - y_1\sin\tilde{\theta}), (1 - 2\tan(\pi/n)\tilde{t}/a)(x_1\sin\tilde{\theta} +$

$y_1\cos\tilde{\theta}), \tilde{b})$ and $\left((1-2\tan(\pi/n)\tilde{t}/a)(x_2\cos\tilde{\theta}-y_2\sin\tilde{\theta}), (1-2\tan(\pi/n)\tilde{t}/a)(x_2\sin\tilde{\theta}+ y_2\cos\tilde{\theta}), \tilde{b}\right)$, respectively. Hence, the line can be described by the following equations:

$$
\begin{cases}
x \in \left(1-\frac{2\tan\left(\frac{\pi}{n}\right)\tilde{t}}{a}\right)\left[\min\left(x_1\cos\tilde{\theta}-y_1\sin\tilde{\theta}, x_2\cos\tilde{\theta}-y_2\sin\tilde{\theta}\right), \max\left(x_1\cos\tilde{\theta}-y_1\sin\tilde{\theta}, x_2\cos\tilde{\theta}-y_2\sin\tilde{\theta}\right)\right] \\
y = \frac{(x_1-x_2)\sin\tilde{\theta}+(y_1-y_2)\cos\tilde{\theta}}{(x_1-x_2)\cos\tilde{\theta}+(y_2-y_1)\mathrm{si}\,\tilde{}}\left(x-\left(1-\frac{2\tan\left(\frac{\pi}{n}\right)\tilde{t}}{a}\right)(x_1\cos\tilde{\theta}-y_1\sin\tilde{\theta})\right)+\left(1-\frac{2\tan\left(\frac{\pi}{n}\right)\tilde{t}}{a}\right)\left(x_1\sin\tilde{\theta}+y_1\cos\tilde{\theta}\right) \\
z = \tilde{b} = \frac{b}{\theta}\tilde{\theta}
\end{cases}
\tag{S3}
$$

By traversing each line corresponding to each $\tilde{t}$ and each $\tilde{\theta}$, a curved shell is formed, which is governed by the following equations:

$$
\begin{cases}
\tilde{t} \in [0, t] \\
\tilde{\theta} \in [0, \theta] \\
x \in \left(1-\frac{2\tan\left(\frac{\pi}{n}\right)\tilde{t}}{a}\right)\left[\min\left(x_1\cos\tilde{\theta}-y_1\sin\tilde{\theta}, x_2\cos\tilde{\theta}-y_2\sin\tilde{\theta}\right), \max\left(x_1\cos\tilde{\theta}-y_1\sin\tilde{\theta}, x_2\cos\tilde{\theta}-y_2\sin\tilde{\theta}\right)\right] \\
y = \frac{(x_1-x_2)\sin\tilde{\theta}+(y_1-y_2)\cos\tilde{\theta}}{(x_1-x_2)\cos\tilde{\theta}+(y_2-y_1)\sin\tilde{\theta}}\left(x-\left(1-\frac{2\tan\left(\frac{\pi}{n}\right)\tilde{t}}{a}\right)(x_1\cos\tilde{\theta}-y_1\sin\tilde{\theta})\right)+\left(1-\frac{2\tan\left(\frac{\pi}{n}\right)\tilde{t}}{a}\right)\left(x_1\sin\tilde{\theta}+y_1\cos\tilde{\theta}\right) \\
z = \tilde{b} = \frac{b}{\theta}\tilde{\theta}
\end{cases}
\tag{S4}
$$

Based on Eq. (S4), a curved shell is determined, then rotated $n-1$ times around the origin at an angle of $2\pi/n$ each time, resulting in the formation of the main structure.

Supplementary Note 2. The reason for attaching stiffeners to the CMCS metamaterial.

Supplementary Figure 2 shows the force-displacement curves and deformation processes of two CMCS metamaterials with or without stiffeners. It can be seen that the middle plane of the latter undergoes a out-of-plane instability during the compression, resulting in a significant drop in plateau phase, which reduces efficiency of energy absorption (EEA). However, this phenomenon does not occur in the former since the stiffeners enhance the vertical stiffness of the rotation plane. Hence, attaching stiffeners to CMCS metamaterials is essential to prevent the reduction of EEA caused by the instability of the middle plane.

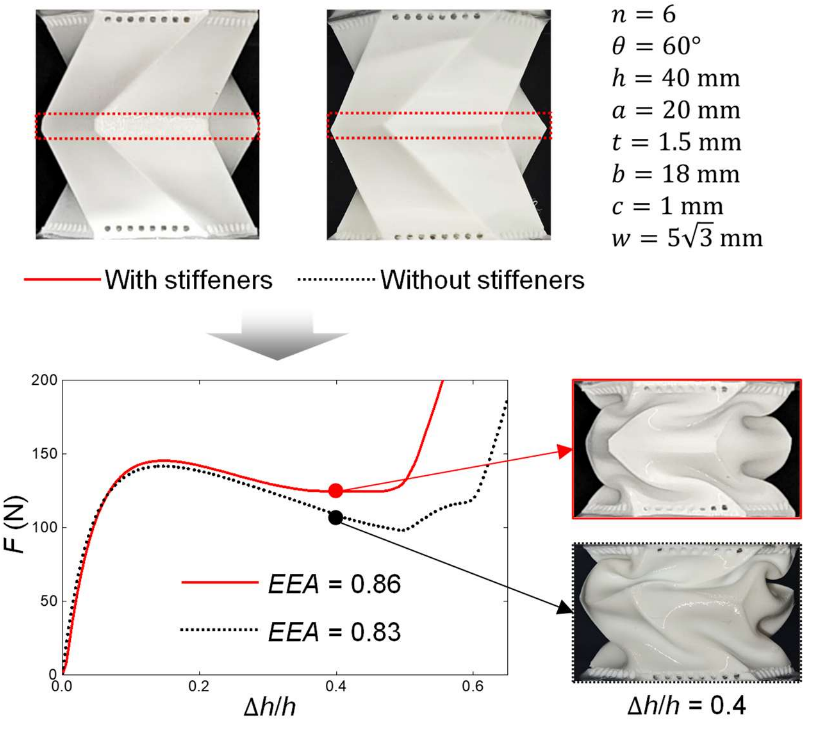

Supplementary Figure 2. Experimental force-displacement curves of CMCS metamaterials with or without stiffeners. The red solid line is the result with stiffeners, and the black dotted line is that without stiffeners; the efficiency of energy absorption (EEA) with or without stiffeners is 0.86 or 0.83, respectively.

Supplementary Note 3. The hexagon, Kresling, and CMCS metamaterials for the comparison.

Supplementary Figure 3 shows the details of the hexagon, Kresling, and CMCS metamaterials. The former two metamaterials are identical to the CMCS metamaterial in terms of n, a, h, c, w, and anticipated mass with 7.75 g, where t can be adjusted to make their anticipated mass equal and the corresponding values of the Kresling and hexagon metamaterials are set to be 1.27 mm and 0.826 mm. The rotation angle of the Kresling metamaterial is also the same with that of the CMCS metamaterial, and the construction way of the Kresling pattern can be found in Supplementary Reference [1]. The two stiffeners of the hexagon metamaterial on the middle plane are moved to bottom and top planes, respectively.

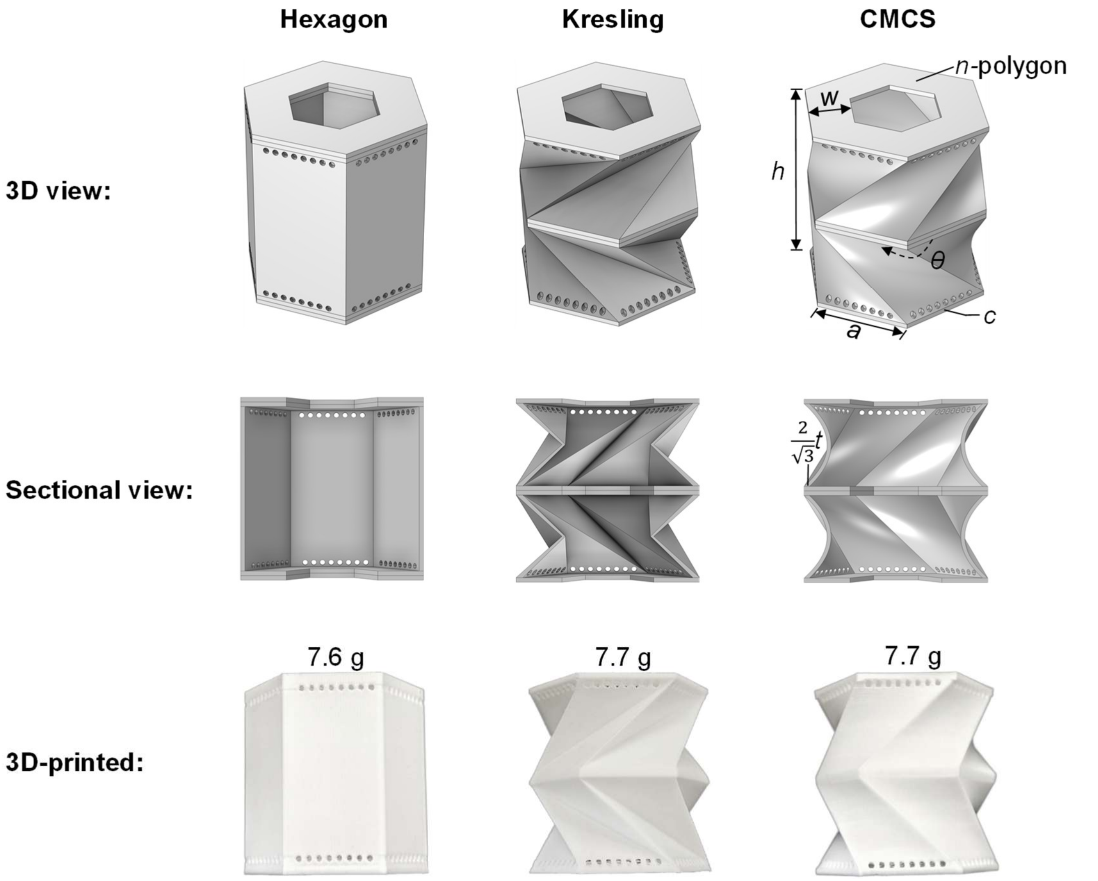

Supplementary Figure 3. Configurations of the hexagon, Kresling, and CMCS metamaterials for the comparison. $t=$ 1 mm, $\theta=$ 60°, $n=$ 6, $h=$ 40 mm, a =20 mm, $c=$ 1 mm, and $w=5\sqrt{3}$ mm.

Supplementary Note 4. Quasi-static compression experiments.

In real engineering applications, energy-absorbing materials are typically sandwiched between a pair of rigid shells, forming the core, and are securely bonded to them. Accordingly, AB glue is used to attach the upper and lower surfaces of a metamaterial to two steel plates, as shown in Supplementary Figure 4. Each steel plate is 50×50×0.6 mm (length × width × thickness). A Universal Testing Machine (UTM) is used to measure force-displacement curves. To simulate quasi-static compression, a loading speed of 0.4 mm/s is applied.

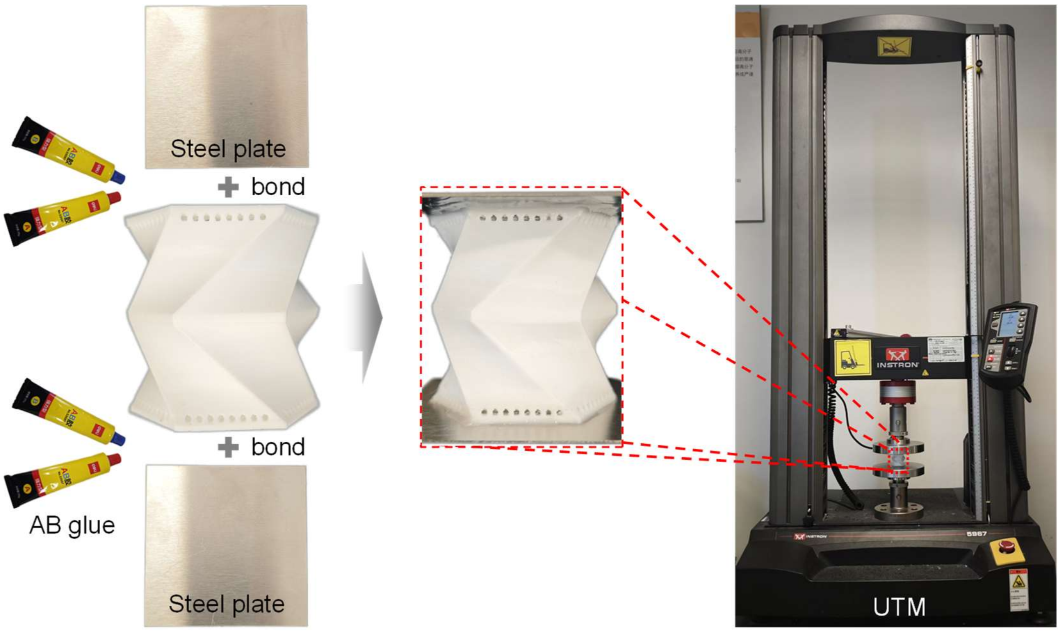

Supplementary Figure 4. Specimen and Universal Testing Machine (UTM) for quasi-static compression experiments.

Supplementary Note 5. Hyperelastic parameters of the TPU material used in 3D-printing.

Hyperelastic Yeoh model is used to describe the nonlinear material behavior of the TPU in 3D-printing, of which function is as follows:

$$W = C_1(I_1 - 3) + C_2(I_1 - 3)^2 + C_3(I_1 - 3)^3 \quad \text{(S5)}$$

where W is the strain energy density; C_1, C_2, and C_3 are the material coefficients; I_1 is the first invariant.

Assume the material is stretched along an axis with a stretch ratio or strain ε. To maintain incompressibility (constant volume), the stretches in the other two perpendicular directions are $\varepsilon^{-\frac{1}{2}}$. Thus, the first invariant I_1 becomes:

$$I_1 = \varepsilon^2 + 2\varepsilon^{-1} \quad \text{(S6)}$$

The Cauchy stress σ in the direction of stretching can be obtained by differentiating the strain energy function with respect to ε:

$$\sigma = \frac{1}{\varepsilon}\left(2\frac{\partial W}{\partial I_1}\frac{\partial I_1}{\partial \varepsilon}\right) \quad \text{(S7)}$$

where

$$\frac{\partial W}{\partial I_1} = C_1 + 2C_2(I_1 - 3) + 3C_3(I_1 - 3)^2 \quad \text{(S8)}$$

$$\frac{\partial I_1}{\partial \varepsilon} = 2\varepsilon - 2\varepsilon^{-2} \quad \text{(S9)}$$

Substitute Eqs. (S6), (S8), and (S9) into the stress expression, namely Eq. (S7), and the stress-strain equation can be obtained as follows:

$$\sigma = 2[C_1 + 2C_2(\varepsilon^2 + 2\varepsilon^{-1} - 3) + 3C_3(\varepsilon^2 + 2\varepsilon^{-1} - 3)^2](1 - \varepsilon^{-3}) \quad \text{(S10)}$$

As shown in Supplementary Figure 5a, a set of tensile and compressive specimens are printed to determine the constitutive parameters of the TPU. Supplementary Figure 5b shows the experimental and fitting stress-strain curves of the 3D-printed specimens with TPU, where $C_1 =$

5.1 MPa, $C_2 = -2.5$ MPa, and $C_3 = 0.8$ MPa.

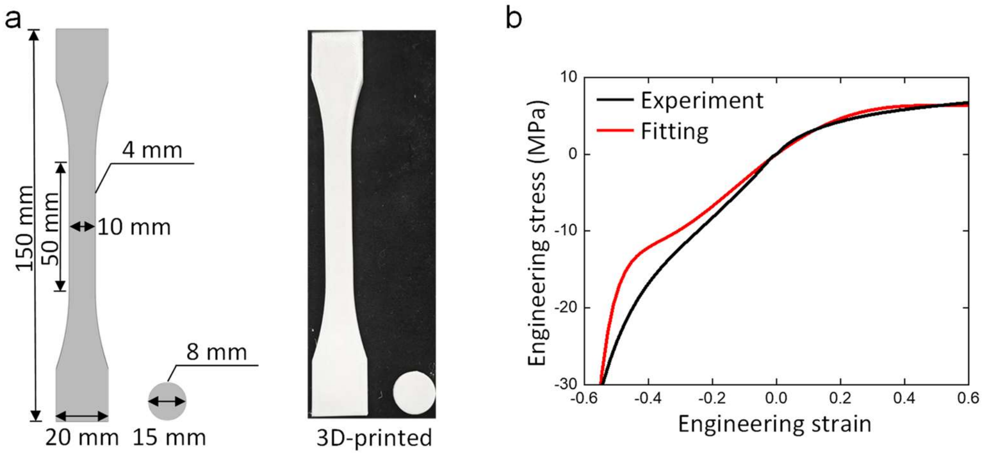

Supplementary Figure 5. Identifying Yeoh model parameters of the TPU material used in 3D-printing. a 3D-printed tensile and compressive specimens with TPU. **b** Stress-strain curves, where the black line is experimental results and the red line is from Yeoh model with $C_1 = 5.1$ MPa, $C_2 = -2.5$ MPa, and $C_3 = 0.8$ MPa.

Supplementary Note 6. Finite element models of the hexagon, Kresling, and CMCS metamaterials.

ABAQUS 2022 is used to build the finite element models of the hexagon, Kresling, and CMCS metamaterials under quasi-static compression. The finite element mesh division of the three metamaterials is given in Supplementary Figure 6a, where the maximum size of elements is lower than 0.5 mm. Eight-node linear brick with reduced integration (element type C3D8R) is adopted for hexahedral elements, and ten-node modified quadratic tetrahedron (element type C3D10M) is used for tetrahedral elements. The lower surface is a fixed boundary condition, and a displacement load is applied to the upper surface. The hyperelastic parameters of their TPU is the same with those in Supplementary Note 5. It can be seen from Supplementary Figure 6b that the force-displacement curves obtained from ABAQUS 2022 are generally consistent with those from experiments. Hence, the results from numerical simulations are credible.

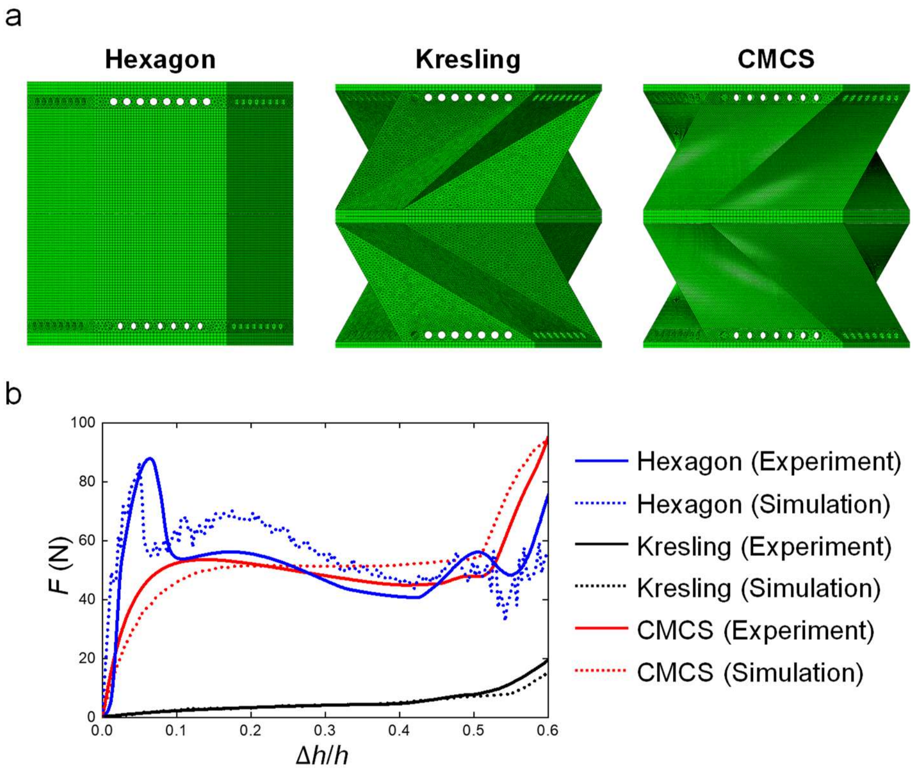

Supplementary Figure 6. Finite element models of the hexagon, Kresling, and CMCS metamaterials under quasi-static compression. a. Finite element mesh division of the hexagon, Kresling, and CMCS metamaterials. **b.** Force-displacement curves by experiments (solid lines) and simulations (dotted lines).

Supplementary Note 7. Experimental force-displacement curves for investigating the relationship of geometry parameters, EEA, and SEA.

Twenty-two of experiments are conducted to discover the influence of geometry parameters, including the thickness t, the side number n, and the rotation angle θ, on force-displacement curves. In these experiments, only one geometry parameter is varied, while all other parameters remain unchanged, where $t =$ 1 mm, $\theta =$ 60°, $n =$ 6, $h =$ 40 mm, a = 20 mm, $b =$ 18 mm, $c =$ 1 mm, and $w = 5\sqrt{3}$ mm. When n varies, the circumcircle of the polygon remains unchanged. All experimental samples, along with their geometry parameters, mass, and relative density, are provided in Supplementary Table 1. In addition, unless otherwise specified, the effective compression area of a microstructure is defined as 1.05 times the side length of the circumscribed square of its circumscribed circle, to prevent collision during compression.

Supplementary Table 1. Information on 3D-printed CMCS metamaterials in Figure 3. h = 40 mm, a = 20 mm, $b =$ 18 mm, $c =$ 1 mm, and $w = 5\sqrt{3}$ mm; the TPU density is 1.19 g/cm^3.

Number	Geometrical parameters			Anticipated mass (g)	Real mass (g)	Mass error (%)	Relative density (%)
	t (mm)	θ (°)	n				
1	1.0	60	6	7.75	7.71	0.52	9.18
2	1.1	60	6	8.14	7.98	1.97	9.5
3	1.2	60	6	8.52	8.45	0.82	10.06
4	1.3	60	6	8.9	8.77	1.46	10.44
5	1.4	60	6	9.29	9.16	1.4	10.91
6	1.5	60	6	9.67	9.4	2.79	11.19
7	1.6	60	6	10.04	9.86	1.79	11.74
8	1.7	60	6	10.42	10.76	3.26	12.81

Number	Geometrical parameters			Anticipated mass (g)	Real mass (g)	Mass error (%)	Relative density (%)
	t (mm)	θ (°)	n				
9	1.8	60	6	10.79	10.92	1.2	13.01
10	1.0	60	4	5.96	6.11	2.52	7.28
11	1.0	60	5	7.08	6.94	1.98	8.27
12	1.0	60	7	8.15	8.05	1.23	9.59
13	1.0	60	8	8.44	8.34	1.18	9.93
14	1.0	30	6	8.35	8.37	0.24	9.97
15	1.0	45	6	8.09	8.06	0.37	9.6
16	1.0	70	6	7.49	7.31	2.4	8.71
17	1.0	72	6	7.43	7.25	2.42	8.63
18	1.0	73	6	7.4	7.21	2.57	8.59
19	1.0	74	6	7.38	7.23	2.03	8.61
20	1.0	75	6	7.34	7.23	1.5	8.61
21	1.0	76	6	7.32	7.2	1.64	8.57
22	1.0	90	6	6.91	7.01	1.45	8.35

Supplementary Note 8. Take the cantilever beam as an example to illustrate that increasing thickness significantly enhances SEA of CMCS metamaterials.

Increasing the thickness t of the CMCS metamaterial will greatly improve its SEA as illustrated in Figure 4a. To explain this phenomenon, we use a cantilever beam as an example, as both the cantilever beam and the curved shell of the CMCS metamaterial have one end fixed and the other end free. As shown in Supplementary Figure 7, the cross section of the cantilever beam is a square with R_1 and R_2, the length is L, a force is vertically applied at the free end, the material is assumed to be linearly elastic, isotropic, and homogeneous, and the small deformation is considered.

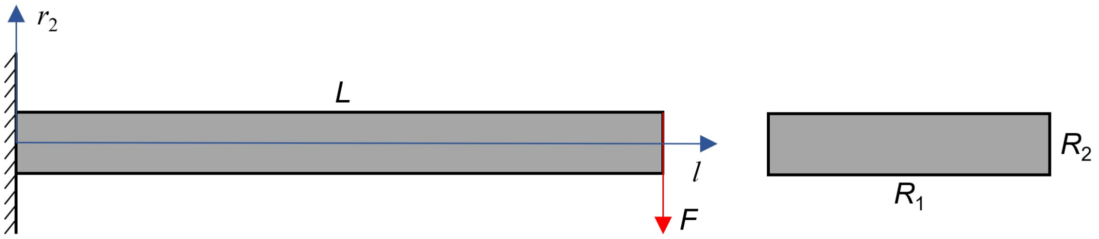

Supplementary Figure 7. Cantilever beam schematic diagram.

The second moment of inertia is as follows:

$$I = \frac{R_1 R_2^3}{12} \tag{S11}$$

The relation of the force and displacement at the free end is the following equation:

$$F(D) = \frac{3EI}{L^3} \tag{S12}$$

where D is the displacement at the free end.

The bending moment on a cross section can be expressed as:

$$M(D, l) = F(D)l \tag{S13}$$

The normal stress in a cross section can be calculated by the following equation:

$$\sigma_1(r_2, D, l) = \frac{M(D,l)r_2}{I} = \frac{3EDlr_2}{L^3} \tag{S14}$$

where r_2 is the absolute distance from the center line of the cross section.

The bending strain energy is as follows:

$$W_1 = \int_0^L \int_{-\frac{R_2}{2}}^{\frac{R_2}{2}} \frac{\sigma_1^2(r_2,D,l)}{2E} R_1 \mathrm{d}r_2 \mathrm{d}l = \frac{ED^2R_1}{8L^3} R_2^3 \tag{S15}$$

Hence, the SEA of the cantilever beam can be expressed as follows:

$$SEA = \frac{W_1}{\rho L R_1 R_2} = \frac{ED^2}{8\rho L^2} R_2^2 \tag{S16}$$

where ρ is the density.

It can be seen from Eq. (S16) that increasing the thickness R_2 can significantly improve the SEA under the same deformation or displacement D. Therefore, increasing the thickness of curved shells will greatly enhance the SEA of CMCS metamaterials.

Supplementary Note 9. The programmability of CMCS metamaterials

As shown in Figure 3, the energy absorption curve of CMCS metamaterials can be controllably tuned by modifying the geometric parameters, demonstrating the inherent programmability of the CMCS design. Supplementary Figure 8 illustrates the parallel programming method for CMCS metamaterials. Two different microstructures are designed and printed, as shown in Supplementary Figure 8a, and then connected in parallel. As demonstrated in Supplementary Figures 8b and c, the energy absorption curve of the parallel configuration closely approximates the sum of the curves of the two individual metamaterials. Additionally, Supplementary Figure 9 presents the matryoshka-like programming method, in which a smaller metamaterial is nested within a larger one. This configuration also yields an energy absorption curve that closely resembles the combined response of the two individual metamaterials. Both design methods demonstrate the high programmability of CMCS metamaterials, enabling greater flexibility in tailoring energy absorption curves and achieving functional customization.

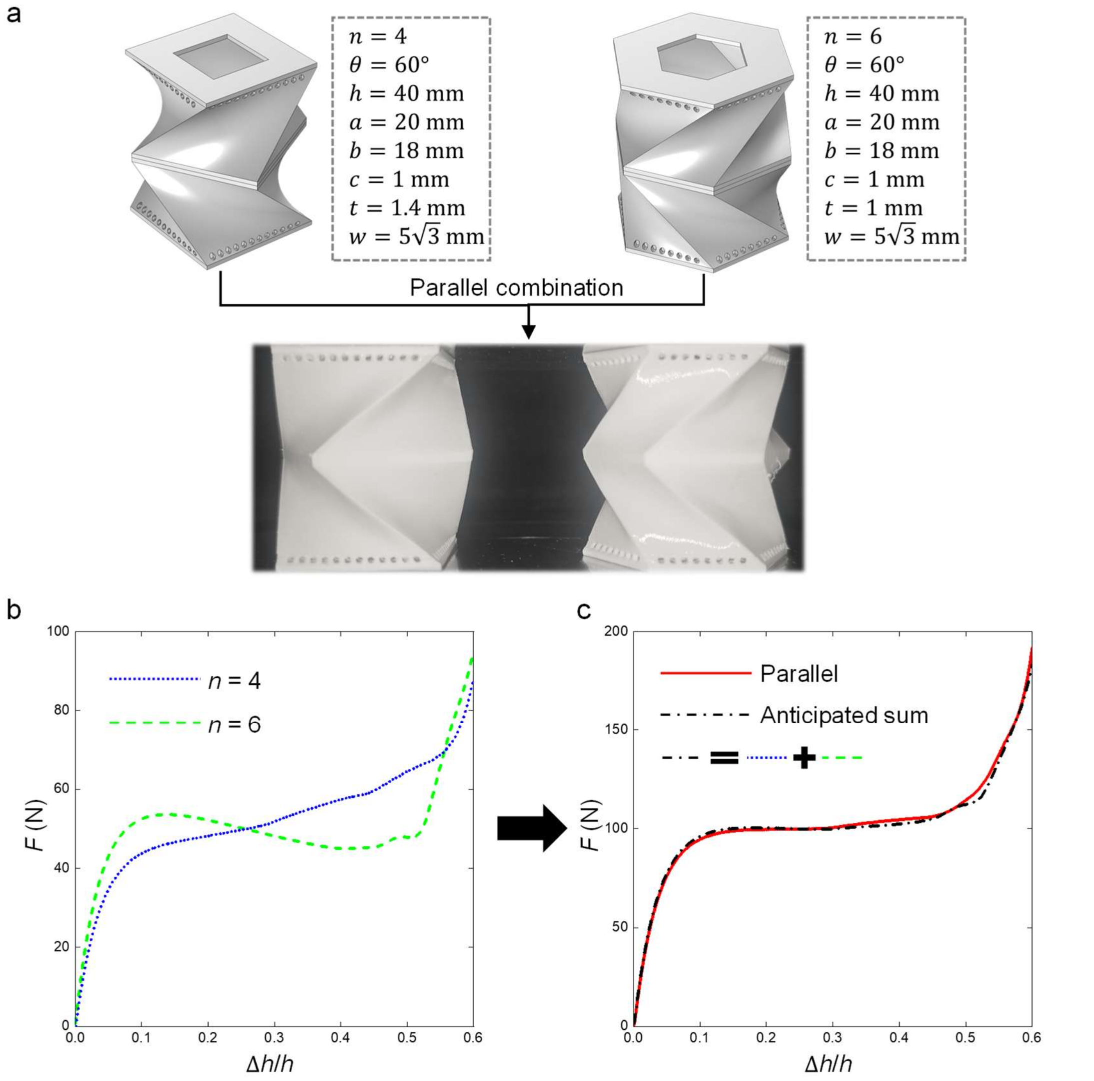

Supplementary Figure 8. Parallel programming method. a. Configurations of the two parallel CMCS metamaterials. **b.** Force-displacement curves of each individual metamaterial under quasi-static compression, where the blue dotted line represents the metamaterial with $n = 4$ and the green dashed line represents that with $n = 6$. **c.** Force-displacement curve of the combined parallel metamaterial under quasi-static compression, where the red solid line represents the actual result of the parallel metamaterial and the dash-dotted line denotes the sum of the two individual curves in Supplementary Figure 8b.

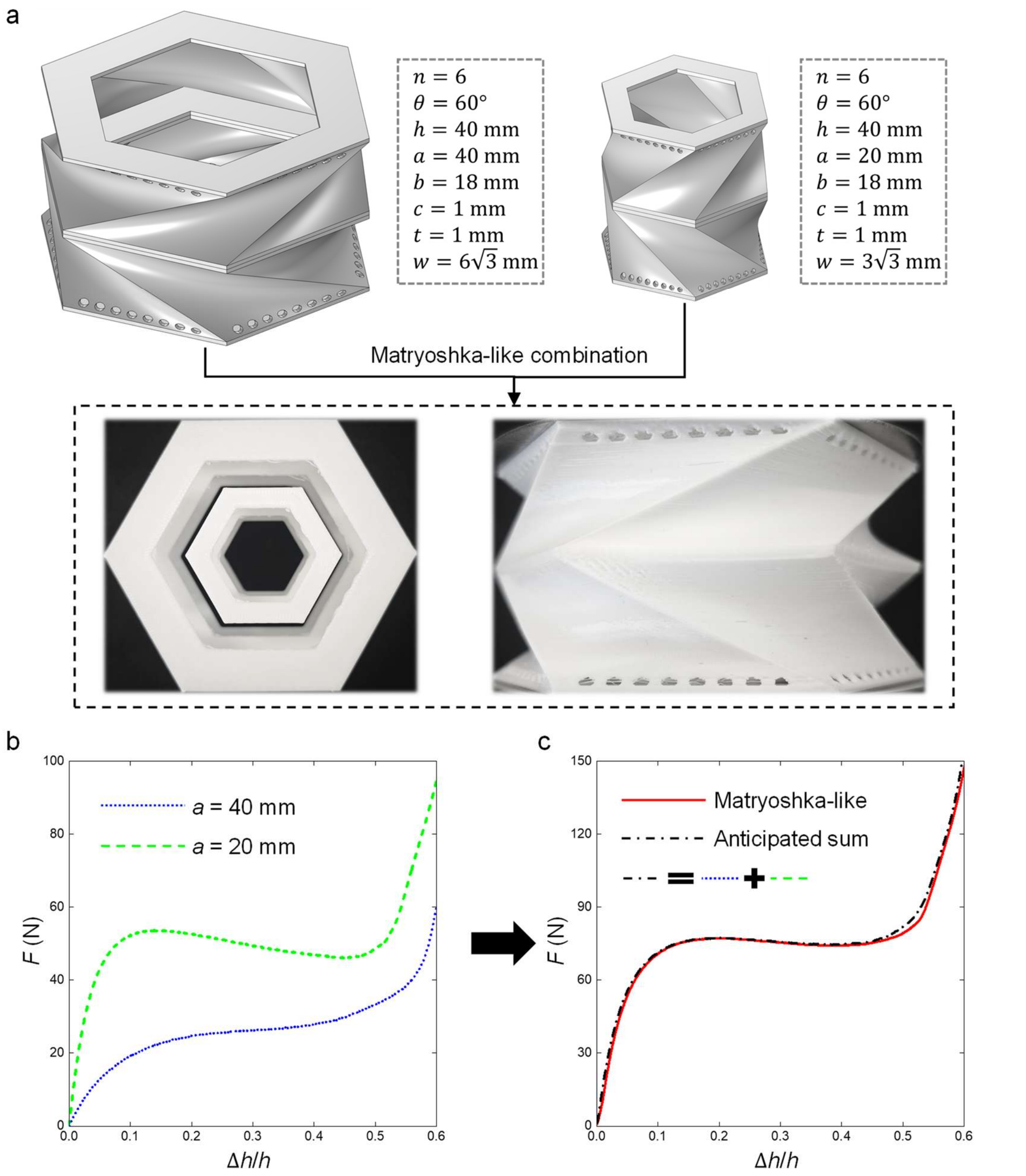

Supplementary Figure 9. Matryoshka-like programming method. a. Configurations of the larger and smaller CMCS metamaterials. **b.** Force-displacement curves of individual metamaterial under quasi-static compression, where the blue dotted line represents the larger metamaterial and the green dashed line represents the smaller one. **c.** Force-displacement curves of the combined matryoshka-like metamaterial under quasi-static compression, where the red solid line represents the actual result of the matryoshka-like metamaterial and the dash-dotted line denotes the sum of the two individual curves in Supplementary Figure 9b.

Supplementary Note 10. The EEA and SEA values for CMCS metamaterials with varying w

To absorb more energy per unit mass while keeping the EEA as high as possible—in other words, to increase the SEA—we discuss the effect of w on the energy absorption curve. Supplementary Figure 10 shows cross-section views of these CMCS metamaterials with varying w, which would be fabricated by 3D-printing technology. Their EEA and SEA values by quasi-static compression experiments are given in Supplementary Table 2.

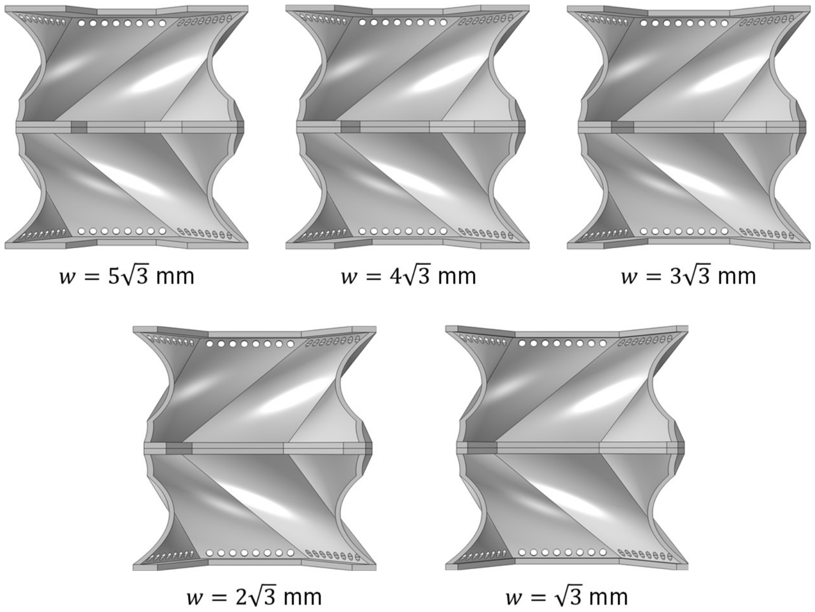

Supplementary Figure 10. Cross-section views of CMCS metamaterials with $w = 5\sqrt{3}, 4\sqrt{3}, 3\sqrt{3}, 2\sqrt{3}$, and, $\sqrt{3}$ mm in Figure 4d. Other geometry parameters, including n, θ, t, h, a, b, and c, are 6, 72°, 1 mm, 40 mm, 20 mm, and 18 mm, respectively.

Supplementary Table 2. EEA and SEA values of CMCS metamaterials in Figure 4d.

w (mm)	Anticipated mass (g)	Real mass (g)	Mass error (%)	Relative density (%)	EEA	SEA (J/kg)
$5\sqrt{3}$	7.43	7.25	2.42	8.63	0.90	65.4
$4\sqrt{3}$	6.88	6.82	0.87	8.12	0.90	69.2
$3\sqrt{3}$	6.24	6.25	0.16	7.44	0.91	75.7
$2\sqrt{3}$	5.5	5.3	3.64	6.31	0.88	83.9
$\sqrt{3}$	4.65	4.7	1.08	5.6	0.87	104.7

Supplementary Note 11. Additional experiments for the Ashby plots

Supplementary Table 3 shows the information on additional experimental CMCS samples for the two Ashby plots of Figures 4f and g.

Supplementary Table 3 Additional experimental CMCS samples for the two Ashby plots of Figures 4f and g. h, b, c, and w are 40 mm, 18 mm, 1 mm, and $5\sqrt{3}$ mm, respectively.

Number	t (mm)	n	θ (deg)	Density (kg/m^3)	EEA	SEA (J/kg)
1	2.4	6	60	182.8	0.81	622
2	3.0	6	60	213.4	0.81	866
3	4.0	4	60	201.4	0.78	729
4	5.0	4	60	235.5	0.76	1000
5	6.0	4	60	267.0	0.77	1506
6	4.0	6	45	277.2	0.79	1661
7	5.0	6	60	303.6	0.84	1491
8	6.0	6	60	341.6	0.80	1835
9	4.0	7	60	271.1	0.84	1215
10	6.0	7	60	361.5	0.80	1825

Supplementary Note 12. Quasi-static compression experiments for 3D lattice CMCS metamaterials.

Supplementary Figure 11 shows the force-displacement of a CMCS metamaterial with 3×3×3 units and its deformation modes at different displacements. Although the periodic 3D lattice metamaterial cannot perfectly preserve the deformation mode of individual units, its force–displacement curve remains relatively smooth throughout the plateau phase and closely matches the anticipant response assuming ideal unit deformation. Meanwhile, the EEA reaches as high as 0.86, demonstrating that the structure maintains highly efficient energy absorption.

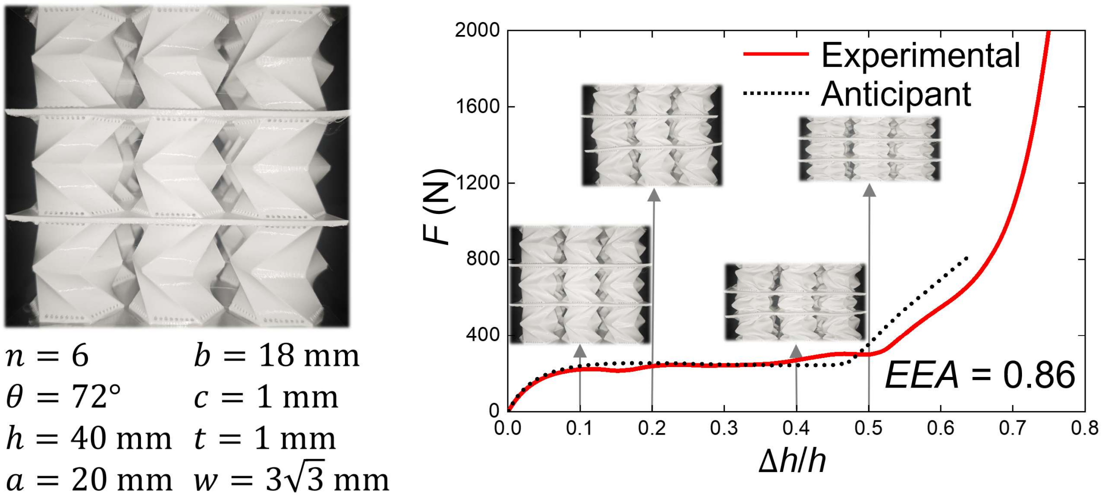

Supplementary Figure 11. Force-displacement curve of a CMCS metamaterial with a 3×3×3 lattice under quasi-static compression. The red solid line is the real experimental curve. The black dotted line is the anticipated curve assuming each unit maintains its ideal deformation mode, which is obtained by multiplying the experimental curve corresponding to the configuration in Figure 4e by a factor of 9.

Supplementary Note 13. The comparison of the CMCS and re-entrant metamaterials.

Supplementary Figures 12a and b show the configurations of the CMCS metamaterial and the re-entrant metamaterial in Figure 5b. For the CMCS metamaterial, the values of n, θ, t, h, a, b, c, and w are 6, 72°, 1.4 mm, 40 mm, 20 mm, 18 mm, 1 mm, and $3\sqrt{3}$ mm, respectively, and its units are arranged square. The center points of each unit are spaced 42 mm apart in pressure surface direction to prevent collisions between adjacent middle planes during compression and rotation. For the re-entrant metamaterial, its shape is illustrated in Supplementary Figure 12b. They are arranged in a triangular pattern on the plumb plane. In addition, two other re-entrant metamaterials, shown in Supplementary Figures 12c and d and featuring different microstructures, have also been printed and tested to further ensure the reliability of the comparison.

The mass of the CMCS metamaterial is 68.6 g, and the re-entrant metamaterials I, II, and III have masses of 55.4 g, 51.7 g, and 52.8 g, respectively. Considering practical engineering applications, metamaterials are typically used as core materials sandwiched between two rigid plates, and the equivalent pressure area is generally defined as the single-connected domain covered by the metamaterial.

Their pressure areas of them are assumed to be 42×42×9 = 15876 mm^2 for the CMCS metamaterial, 42×123.2 = 5174.4 mm^2 for the re-entrant metamaterial I, 42×115.5 = 4851 mm^2 for the re-entrant metamaterial II, and 42×123 = 4972.8 mm^2 for the re-entrant metamaterial III. The relative densities of the four metamaterials are 9.1%, 22.5%, 22.4%, and 22.3%, respectively. Supplementary Tables 4 and 5 validate the quality of the 3D-printed CMCS and re-entrant metamaterials, respectively.

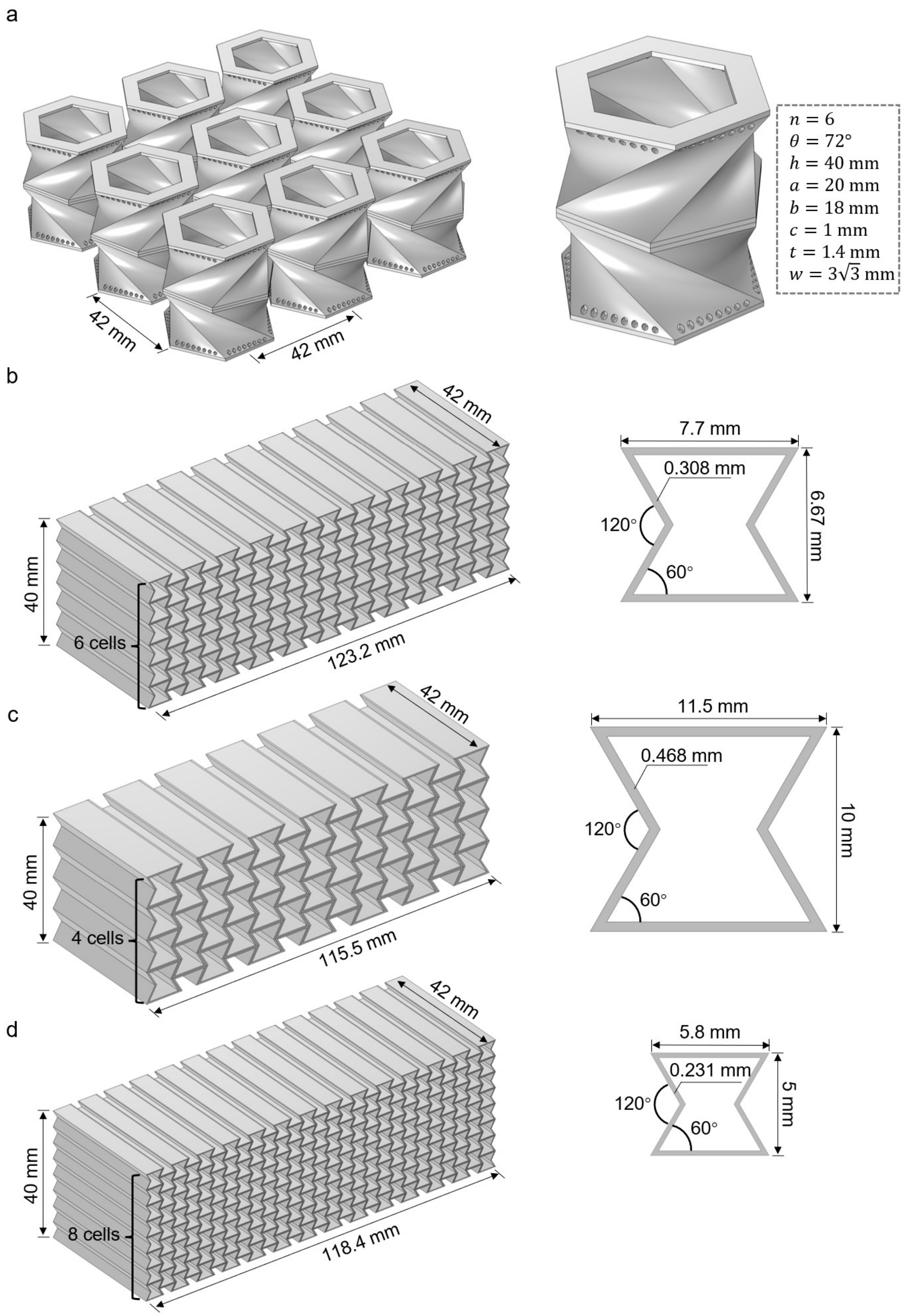

Supplementary Figure 12. Configurations of metamaterials in Figure 5b. a. The CMCS metamaterial. **b.** The re-entrant metamaterial I. **c.** The re-entrant metamaterial II. **d.** The re-entrant metamaterial III.

Supplementary Table 4 Validating the quality of the 3D-printed CMCS metamaterial. The measured value is shown on the left and the anticipated value on the right in each cell (measured | anticipated).

Sample	Mass (g)	h (mm)	a (mm)	b (mm)
CMCS metamaterial	68.6 | 69.0	39.99 | 40.00	20.01 | 20.00	18.02 | 18.00

Supplementary Table 5 Validating the quality of the 3D-printed re-entrant metamaterials. The measured value is shown on the left and the anticipated value on the right in each cell (measured | anticipated).

Sample	Mass (g)	Height (mm)	Length (mm)	Width (mm)
Re-entrant metamaterial I	55.4 | 56.3	40.02 | 40.00	123.11 | 123.20	41.98 | 42.00
Re-entrant metamaterial II	51.7 | 52.9	40.01 | 40.00	155.45 | 155.50	42.01 | 42.00
Re-entrant metamaterial III	52.8 | 54.3	40.01 | 40.00	118.32 | 118.40	41.99 | 42.00

Supplementary Figure 13a shows the force-displacement curves of the four metamaterials, where the force is normalized by dividing it by the area. It can be seen that they exhibit similar plateau forces per unit area, making them suitable for the same protective scenarios and have the fair comparison. EEA, SEA, plateau phase, densification displacement, and ULC for the CMCS and re-entrant metamaterials are compared in Supplementary Figure 13b, of which values are provided in Supplementary Table 6. Obviously, the energy absorption metrics of the three re-entrant metamaterials are lower than those of the CMCS metamaterial, with the SEA of the CMCS metamaterial being nearly three times higher.

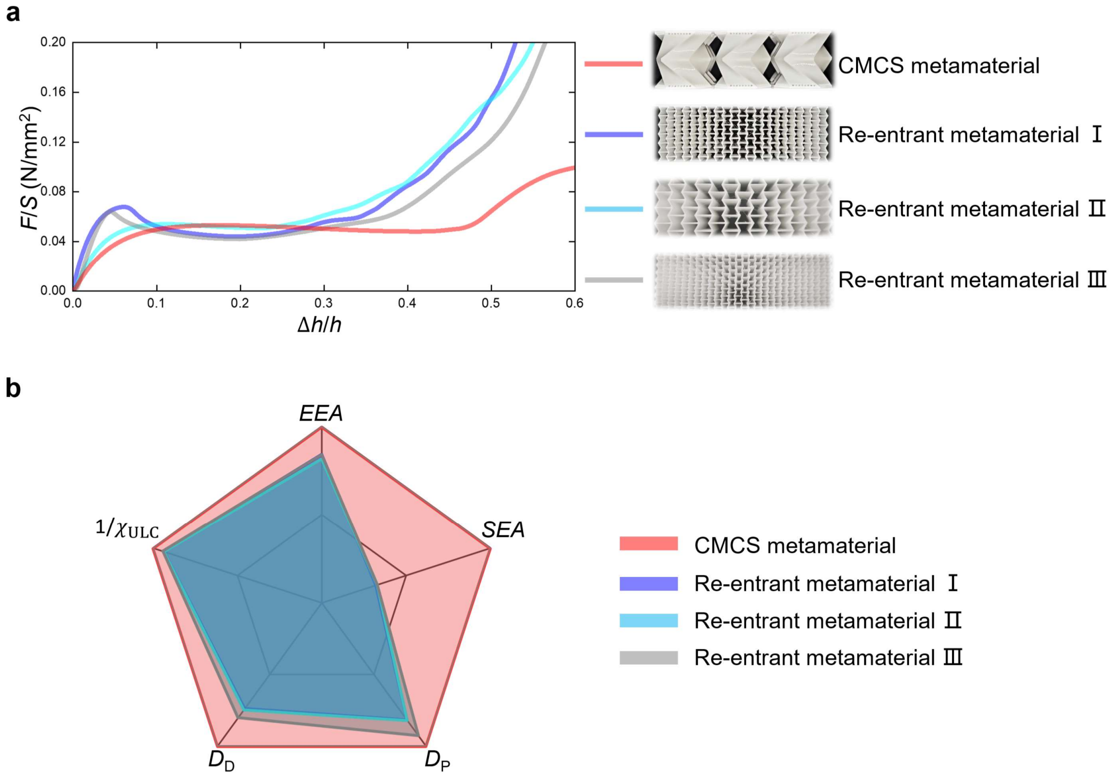

Supplementary Figure 13. Comparison results of the four metamaterials. a. Force-displacement curves, where S is the pressure area. **b.** Radar chart displays EEA, SEA, plateau phase D_{P}, densification displacement D_{D}, and undulation of load-carrying (ULC) χ_{ULC}.

Supplementary Table 6. EEA, SEA, plateau phase, densification displacement, and ULC values for the CMCS and re-entrant metamaterials.

Energy absorption metric	CMCS metamaterial	Re-entrant metamaterial I	Re-entrant metamaterial II	Re-entrant metamaterial III
EEA	0.88	0.74	0.72	0.74
SEA (J/kg)	200	64	66	66
D_{P} (mm)	14.08	11.52	11.56	13.08
D_{D} (mm)	18.52	13.68	13.84	14.88
χ_{ULC}	0.130	0.139	0.139	0.138

In addition, Supplementary Figure 14 compares the variation in SEA of CMCS and re-entrant metamaterials with increasing relative density. For CMCS metamaterials, the thickness varies from 1.0 mm to 6.0 mm, with other geometric parameters consistent with those in Supplementary Figure 3. These data are sourced from the Ashby plot of Figure 4g. For re-entrant metamaterials, the thickness values are 0.308 mm, 0.35 mm, and 0.4 mm, with other geometric parameters identical to those of re-entrant metamaterial I shown in Supplementary Figure 12b. It can be seen that the growth trend of CMCS metamaterials is significantly steeper and consistently higher than that of the re-entrant metamaterials. Therefore, it is reasonable to infer that CMCS metamaterials are superior to re-entrant metamaterials in SEA.

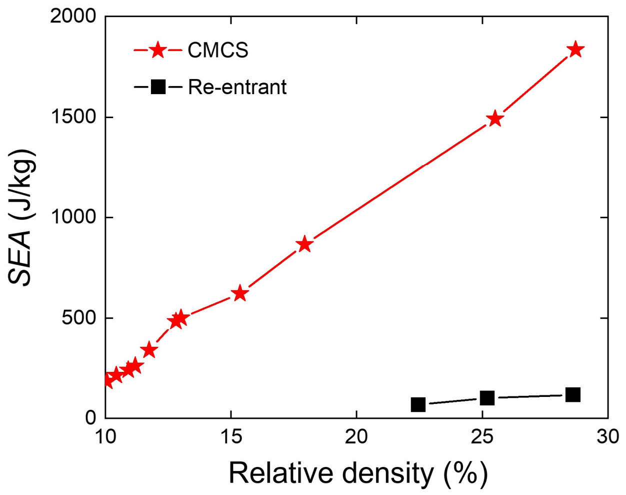

Supplementary Figure 14. Comparison of SEA trends for CMCS and re-entrant metamaterials with increasing relative density. For CMCS metamaterials, the thickness varies from 1.0 mm to 6.0 mm, with other geometric parameters consistent with those in Supplementary Figure 3. For re-entrant metamaterials, the thickness values are 0.308 mm, 0.35 mm, and 0.4 mm, with other geometric parameters identical to those of re-entrant metamaterial I shown in Supplementary Figure 12b.

Supplementary Note 14. Drop hammer test.

As illustrated in Supplementary Figure 15, the drop hammer machine system is mainly composed of a gripper, a pair of slide rails, a hammer body, a hammer head, a sensor, a platform, a main console, and a data acquisition system. To simulate practical protective drop scenarios, the printed CMCS metamaterial is affixed to the bottom of the hammer head using double-sided tape, as shown in Supplementary Figure 16. When either the CMCS metamaterial or the hammer head contacts the platform, an accelerometer records the hammer's response. The hammer has a mass of 1 kg, and the drop height—measured from the bottom of the CMCS metamaterial to the top of the platform—is set to be 1.0 m. Twenty identical drop tests are conducted on the CMCS metamaterial to evaluate its reusability.

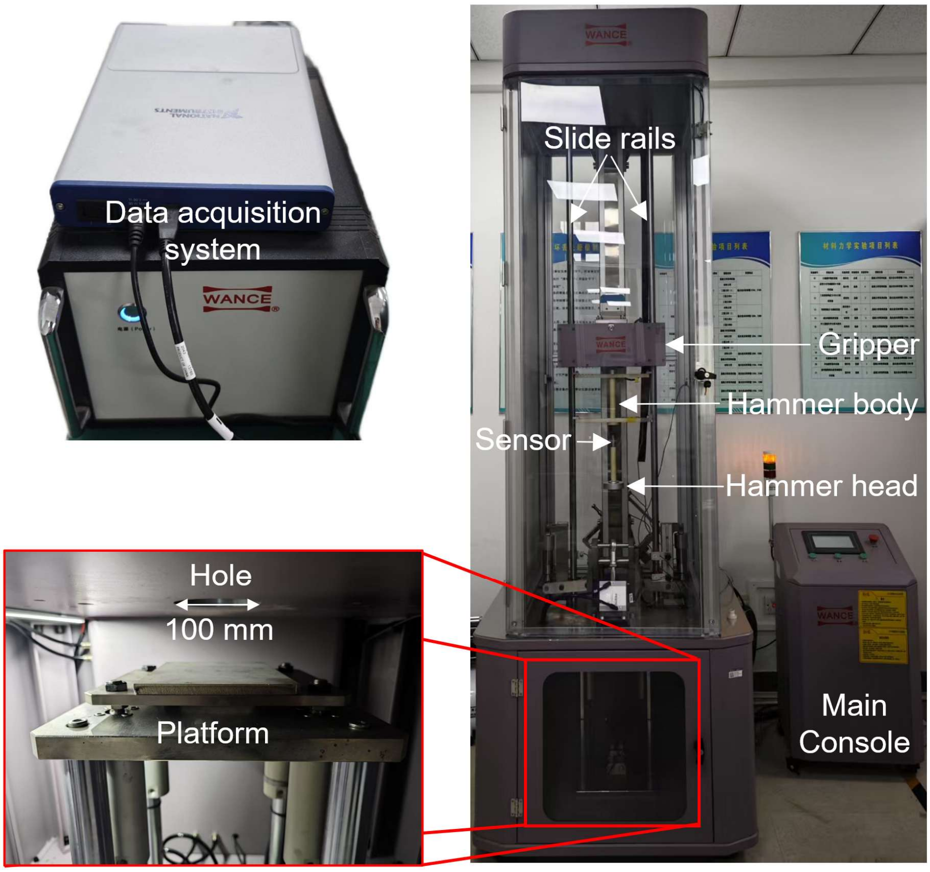

Supplementary Figure 15. The drop hammer machine system. It is mainly composed of a gripper, a pair of slide rails, a hammer body, a hammer head, a sensor, a platform, a main console, and a data acquisition system. Additionally, a computer is used for data display and process.

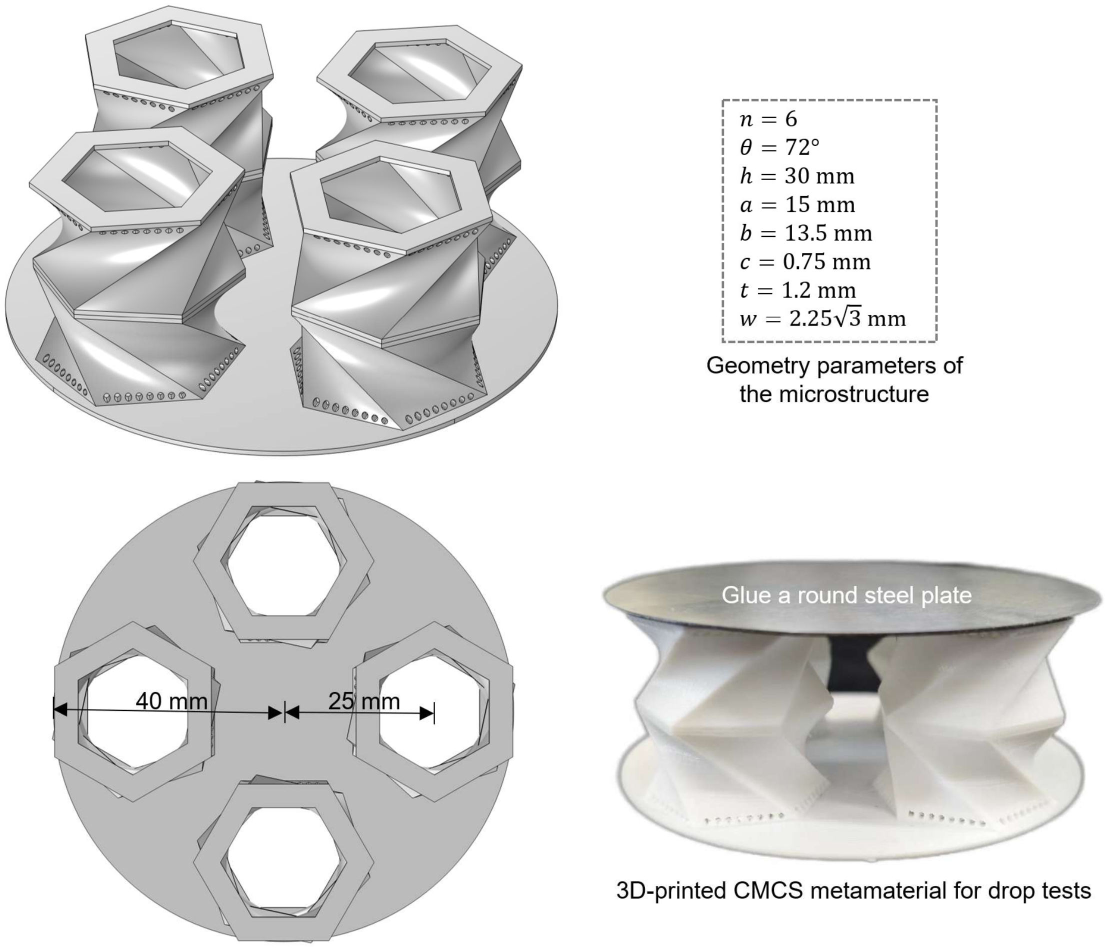

Supplementary Figure 16. The CMCS metamaterial for drop tests. Its shape is proportionally scaled down to 75% of that in Figure 4e, except for the thickness t, and its mass is 16.5 g. A round steel plate is affixed to the impact face, of which diameter and thickness are 80 mm and 0.6 mm, respectively.

Supplementary Note 15. Potential applications

The CMCS metamaterials composed of TPU offer notable advantages, including lightweight design, reusability, and exceptional flexibility. These properties make them highly adaptable for a wide range of applications. For instance, they can serve as protective layers for buildings or armored vehicles, effectively mitigating blast damage, as shown in Supplementary Figure 17a. Additionally, their reusability and flexibility enable them to be fabricated into safety helmets, as illustrated in in Supplementary Figure 17b, providing enhanced protection for individuals. Furthermore, their lightweight nature makes them ideal for use as protective structures for drones, as detailed in in Supplementary Figure 17c, safeguarding blades and precision components from impact-related damage.

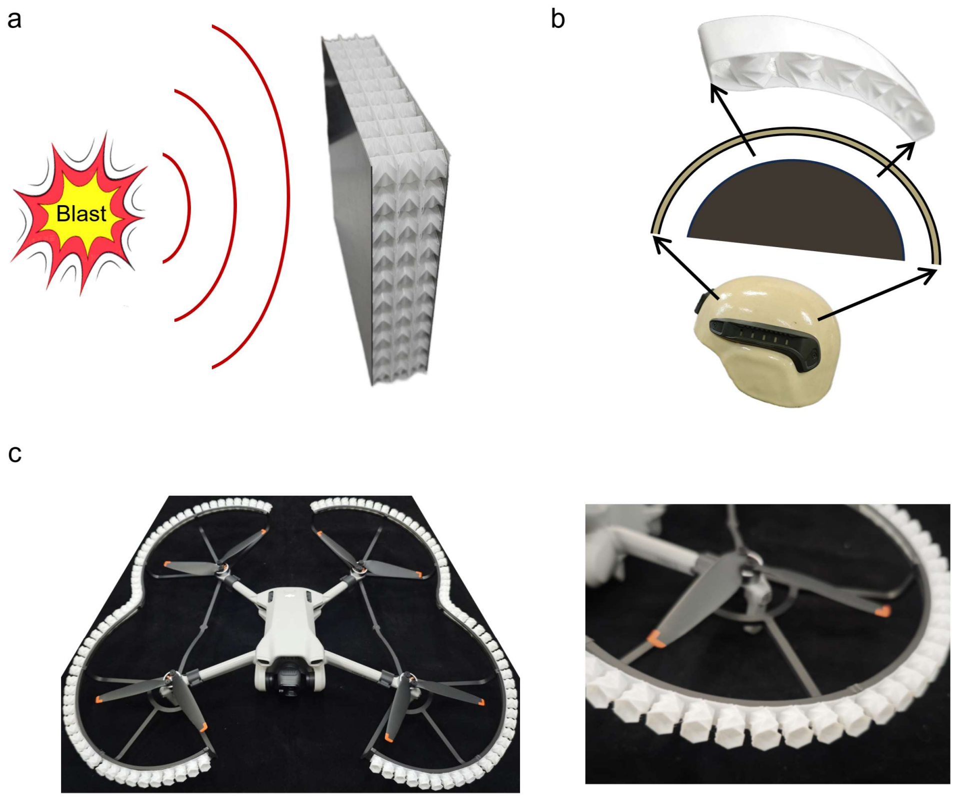

Supplementary Figure 17. Potential applications of CMCS metamaterials. a. Protective layers for buildings or armored vehicles. **b.** Suspension system for safety helmets (this figure is created in PowerPoint). **c.** Energy-absorbing structures for drones.

In addition, Supplementary Figure 18 illustrates a gradient design of the CMCS metamaterial, which shows potential for application in aircraft landing-gear cushioning structures. Owing to its high load-bearing capacity and reusability, the CMCS metamaterial is a promising candidate for integration into such cushioning structures. Moreover, its inherent programmability allows the creation of graded plateau phases, enabling the cushioning system to adapt to varying landing scenarios, such as normal landings, hard landings, and emergency reserves, thereby providing tailored and reliable energy-absorption performance.

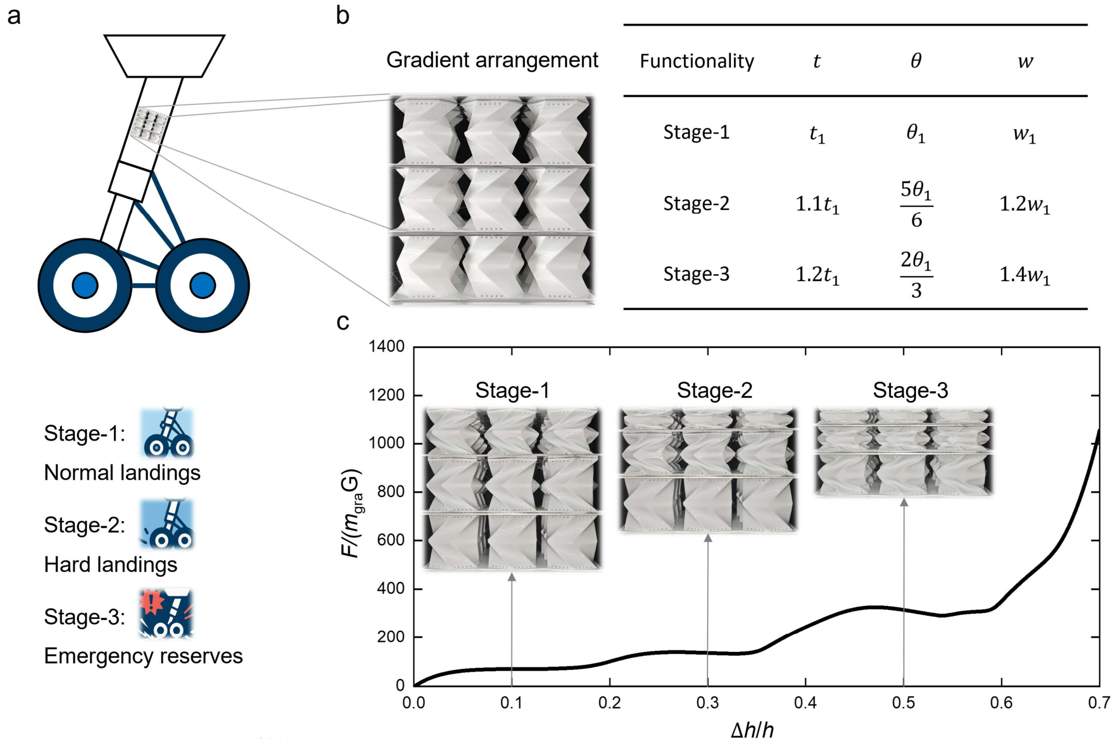

Supplementary Figure 18. Schematic illustration of the potential application of the gradient CMCS metamaterial to aircraft landing-gear cushioning structures. a. Aircraft landing gear (this figure is created in PowerPoint). **b.** Gradient design of the CMCS metamaterial. **c.** Experimental force-displacement curve, where m_{gra} is the mass of the gradient CMCS metamaterial and G is the gravity.

Supplementary References

[1] Liu, C. X., Wang, X., Liu, W., Yang, Y. F., Yu, G. L., & Liu, Z. (2024). A physics-informed neural network for Kresling origami structures. *International Journal of Mechanical Sciences*, *269*, 109080.
