# Supplementary material for: Chiral multi-curved shell metamaterials integrating compression-torsion and buckling mechanisms for ideal energy absorption
Source: Nat Commun. 2025 Dec 12;16:11359. doi: 10.1038/s41467-025-66443-y (PMC12727706; doi:10.1038/s41467-025-66443-y)
Supplement: Supplementary file 2 — Description of Additional Supplementary Files [file 41467_2025_66443_MOESM2_ESM.pdf]

Description of Additional Supplementary Files

File Name: Supplementary Movie 1

Description: Quasi-static compression experiment of the CMCS microstructure in Figure 1b.

File Name: Supplementary Movie 2

Description: Quasi-static compression experiment of the periodic CMCS metamaterial in Figure 5b.

File Name: Supplementary Movie 3

Description: Quasi-static compression experiment of the re-entrant metamaterial in Figure 5b.
